# White matter microstructure in transmasculine and cisgender adolescents: A multiparametric and multivariate study

**DOI:** 10.1371/journal.pone.0300139

**Published:** 2024-03-12

**Authors:** Lindsey T. Thurston, Malvina N. Skorska, Nancy J. Lobaugh, Kenneth J. Zucker, M. Mallar Chakravarty, Meng-Chuan Lai, Sofia Chavez, Doug P. VanderLaan

**Affiliations:** 1 Department of Psychology, University of Toronto Mississauga, Mississauga, Ontario, Canada; 2 Campbell Family Mental Health Research Institute, Centre for Addiction and Mental Health, Toronto, Ontario, Canada; 3 Brain Health Imaging Centre, Centre for Addiction and Mental Health, Toronto, Ontario, Canada; 4 Division of Neurology, Temerty Faculty of Medicine, University of Toronto, Toronto, Ontario, Canada; 5 Department of Psychiatry, Temerty Faculty of Medicine, University of Toronto, Toronto, Ontario, Canada; 6 Cerebral Imaging Centre, Douglas Mental Health University Institute, Montreal, Ontario, Canada; 7 Department of Psychiatry, The Hospital for Sick Children, Toronto, Ontario, Canada; 8 Autism Research Centre, Department of Psychiatry, University of Cambridge, Cambridge, United Kingdom; University of Rochester, UNITED STATES

## Abstract

Adolescence is a sensitive developmental period for neural sex/gender differentiation. The present study used multiparametric mapping to better characterize adolescent white matter (WM) microstructure. WM microstructure was investigated using diffusion tensor indices (fractional anisotropy; mean, radial, and axial diffusivity [AD]) and quantitative T1 relaxometry (T1) in hormone therapy naïve adolescent cisgender girls, cisgender boys, and transgender boys (i.e., assigned female at birth and diagnosed with gender dysphoria). Diffusion indices were first analyzed for group differences using tract-based spatial statistics, which revealed a group difference in AD. Thus, two multiparametric and multivariate analyses assessed AD in conjunction with T1 relaxation time, and with respect to developmental proxy variables (i.e., age, serum estradiol, pubertal development, sexual attraction) thought to be relevant to adolescent brain development. The multivariate analyses showed a shared pattern between AD and T1 such that higher AD was associated with longer T1, and AD and T1 strongly related to all five developmental variables in cisgender boys (10 significant correlations, *r* range: 0.21–0.73). There were fewer significant correlations between the brain and developmental variables in cisgender girls (three correlations, *r* range: -0.54–0.54) and transgender boys (two correlations, *r* range: -0.59–0.77). Specifically, AD related to direction of sexual attraction (i.e., gynephilia, androphilia) in all groups, and T1 related to estradiol inversely in cisgender boys compared with transgender boys. These brain patterns may be indicative of less myelination and tissue density in cisgender boys, which corroborates other reports of protracted WM development in cisgender boys. Further, these findings highlight the importance of considering developmental trajectory when assessing the subtleties of neural structure associated with variations in sex, gender, and sexual attraction.

## Introduction

Transgender is an adjective and umbrella term that describes a person whose gender identity is different from their sex assigned at birth [1]. Transgender individuals over the age of 15 years approximate 0.33% of the Canadian population and almost 0.8% of youth aged 15 to 24 years [2]. Recent studies suggest transgender boys (i.e., assigned female at birth) make up a greater proportion of the transgender adolescents accessing gender-affirming care services [3, 4]. Despite this proportion of transgender youth, a comprehensive understanding of neural development in transgender adolescents is lacking, particularly before the initiation of gender-affirming hormone therapy (GAHT). Gender-affirming medical care may include puberty suppression and exogenous sex steroid hormones to assist with physical and psychological gender affirmation [5], hereafter referred collectively as GAHT. In cisgender youth, pubertal stage and sex steroid hormones relate to the development of WM microstructure [6]. As such, it is possible that GAHT contributes to neural development in transgender youth either through suppression of pubertal development (e.g., [7]) or addition of exogenous hormones (e.g., [8]).

White matter (WM) microstructure, defined as the organization of axons and degree of myelination, establishes neural connectivity between functionally related structures [9]. WM microstructure development varies with sex assigned at birth [10] and gender socialization [11]. The independent influence of biological and social factors on neural phenotype is difficult to disentangle; therefore, the composite term sex/gender is used to refer to the complex synthesis of sex assigned at birth and gender socialization [12]. Sex/gender differences in WM microstructure may be partially attributed to differences in pubertal development and sex steroid hormones [6, 13–15]. Because sex steroid hormones are also posited to promote aspects of psychosexual development such as gender identity and sexual orientation [16], it is possible that WM microstructure also differs in relation to these aspects of psychosexuality. Indeed, in certain brain regions, WM microstructure differs with gender identity in studies of transgender adults [17], but studies in transgender adolescents are limited. Thus, characterizing WM microstructure development in transgender boys prior to starting GAHT is integral to building a foundation from which to elucidate the progression of physiological and psychological outcomes of GAHT.

WM microstructure can be assessed *in vivo* with diffusion weighted imaging techniques. Diffusion tensor imaging (DTI) measures the rate and direction of water diffusion in the brain and, therefore, is sensitive to tissue composition (e.g., myelination) and organization (i.e., fiber orientation [9]). Developmental diffusion weighted imaging studies have demonstrated that axonal organization, myelination, and packing of WM tissue increase with neural development (for review, see [10]). Mature WM microstructure is characterized by high directionality and organization, quantified by high fractional anisotropy (FA), and low mean diffusivity (MD) [18]. Axial diffusivity (AD; i.e., the diffusion parallel to the axon) and radial diffusivity (RD; i.e., the diffusion perpendicular to the axon) are more specific indices that can offer proxy measurements for axonal tortuosity and organization [19], and degree of myelination, respectively [10, 20, 21]. Although less commonly reported, AD and RD have also shown sex/gender differentiation in adolescents [22–24]. MD and FA may allow for only limited inferences of tissue composition [25]; thus, including AD and RD in analyses improves the interpretation of WM tissue composition [25].

Further, characterization of WM tissue composition can be aided via multiparametric mapping, which could include the more specific diffusion metrics, AD and RD, as well as other myelin-sensitive imaging methods, such as T1 relaxation time mapping [26, 27]. T1 maps quantify the presence and interaction of water molecules and macromolecules, such as lipids and proteins, in neural tissues. Myelin can influence the length of T1. The presence of macromolecules and fewer free-water molecules (i.e., water that is not in the myelin sheath) in WM results in shorter T1 values compared with gray matter [27]. Furthermore, T1 relaxation times shorten with age potentially due to increased myelination [26].

The development of WM microstructure follows an earlier trajectory in adolescent cisgender girls than boys (i.e., [22, 24, 28]), although some studies report no sex/gender differences in development (i.e., [23, 29]; for review, see [10]). Therefore, it has been recommended that pubertal development stage should be measured in adolescent DTI studies, in addition to age [6, 30]. Despite the protracted development in adolescence, cisgender men typically have greater FA and lower MD than cisgender women in adulthood [31, 32].

Sex/gender differences in WM microstructure and its development are reportedly partly influenced by sex steroid hormones [6, 32]. Testosterone has been related to indices of WM microstructure diffusion in both adolescent cisgender boys and girls (e.g., [29]), but the relationship with estradiol is less often investigated [6]. In pubertal transgender boys naïve to GAHT, the endogenous concentration of estradiol likely exceeds that of testosterone; thus, focusing on the potential influence of estradiol on WM microstructure is most pertinent. In cisgender adolescents, estradiol related positively [29, 30] and negligibly [29, 33] to WM microstructure organization and diffusion.

Studies of transgender adults prior to using GAHT show inconclusive findings. Studies have reported that WM microstructure in transgender adults is significantly different from the sex assigned at birth-matched comparison group but similar to the gender-matched comparison group (transgender men: [34]; transgender women: [35]); intermediate between sex assigned at birth and gender (transgender women: [36]; transgender men and women: [37]); and similar to the sex assigned at birth-matched comparison group but different from the gender-matched group (transgender men: [35]). The inconsistent directionality makes it challenging to infer an influence of sex steroid hormones in transgender individuals before GAHT. Still, further investigation is warranted given that some findings corroborate a potential role for sex steroid hormones in the development of sex/gender-related differences in WM microstructure [8].

One study, assessing WM microstructure in transgender adolescents using pubertal suppression GAHT, reported lower FA bilaterally in the IFOF, forceps major, and corpus callosum in the transgender adolescents using pubertal suppression GAHT compared with the cisgender adolescents not using GAHT [7]. In contrast, WM microstructure was similar between pre-pubertal cisgender children and transgender children not using GAHT [7]. Thus, the lower FA in transgender adolescents using pubertal suppression GAHT may suggest that pubertal development and/or sex steroid hormones influence WM microstructure development. Although this study provides a valuable starting point for transgender adolescent DTI research, the lack of a hormone therapy-naïve transgender adolescent group confounds the interpretations of the findings and highlights the need to assess WM microstructure development prior to any GAHT use.

Across the DTI literature in transgender populations, the measurement of sexual orientation is inconsistent (e.g., not considered [7, 37, 38], included homogenous sexual orientations [34, 36]), making it unclear whether observed WM microstructure differences are driven by gender identity and/or sexual orientation [17]. Burke et al. [35] did include a range of sexual identities in their cisgender and transgender adult groups and found that WM microstructure was not influenced by sexual orientation in the transgender groups [35]. However, one adolescent study showed that cortical structure correlated with the strength and direction of sexual attraction in masculine cisgender and transgender adolescents [39]. Therefore, sexual orientation may be important for characterizing neural phenotype in WM research.

The present study assessed WM microstructure in cisgender girls, cisgender boys, and transgender boys using a mass-univariate approach (i.e., tract-based spatial statistics [TBSS]) and a multivariate approach (i.e., partial least squares [PLS]) to incorporate additional metrics of WM microstructure. The study aimed to better characterize adolescent WM and underlying tissue composition in these groups using multiparametric mapping to understand the influence of pubertal development and sex steroid hormones on WM microstructure.

The diffusion indices FA, MD, AD, and RD were first assessed using the mass-univariate TBSS analysis for group comparisons. Any diffusion index found to be significantly different across groups was then included in two PLS analyses. The PLS analyses also included T1 relaxation time mapping as a complementary, multiparametric measure of WM tissue composition. Given the neurodevelopmental literature on adolescence indicating protracted WM microstructure development in cisgender boys than girls [10, 23, 24, 28], it was hypothesized that axonal organization and myelination as measured by FA, MD, AD, RD, and T1 relaxation time would be stronger/larger in cisgender girls than boys. Transgender boys were predicted to be intermediate to cisgender girls and boys based on the findings from transgender adults [36, 37].

The second PLS analysis correlated selected WM microstructure metrics with five variables noted as relevant to adolescent brain development. Three of these variables were related to pubertal development, including age, physical development during puberty as measured by the Pubertal Development Scale (PDS [40]), and serum estradiol concentration. Two sexual attraction variables (i.e., strength, direction of attraction) were also included to index psychosexual development. Overall, variables related to pubertal development were hypothesized to relate positively to WM microstructure organization and myelination [18]. Age, physical development, and strength of sexual attractions were predicted to relate positively with WM maturation, as characterized by greater tissue organization and myelination [6], and estradiol concentration was predicted to relate positively to myelination (e.g., [41–43]). Direction of sexual attraction was predicted to relate to T1 relaxation time such that stronger gynephilic attractions would relate to shorter T1, as found in Skorska et al. [39] for cortical gray matter.

## Methods

### Participants

Eligible adolescents (*N* = 51) participated in a larger neuroimaging study. Data from five participants were removed from the present analysis due to image processing errors (*n* = 1) and excessive movement during scanning (*n* = 4). Thus, this study included 46 individuals aged 12 to 17 years (mean = 15.4 years; standard deviation, [SD] = 1.66). Structural and functional magnetic resonance imaging (MRI) data from a largely overlapping cohort of these participants have previously been published [39, 44]; the WM metrics reported here have not yet been published. Data were collected from 2014 to 2018 at the Centre for Addiction and Mental Health (CAMH) in Toronto, Canada. As this research involved in-person data collection, researchers had access to participant identifying information. Adolescent participants were grouped into cisgender girls (assigned female at birth; *n* = 17), cisgender boys (assigned male at birth; *n* = 14), and transgender boys, including gender-questioning adolescents (assigned female at birth; *n* = 15). The majority (69.5%) of participants reported their ethnicity as “European”/ “White” (see summary in S1 Table).

Transgender youth were eligible to participate if they had received a formal diagnosis of gender dysphoria, a clinical diagnosis of the distress associated with having a gender identity that is different from the sex assigned at birth [1]. Exclusion criteria for all participants included receiving hormone therapy (i.e., GAHT) apart from hormonal contraception, a known hormonal or disorder of sex development, contraindications to MRI, oral orthodontics, or head trauma. Additional exclusion criteria for cisgender participants included previous mental health diagnoses, inclusion in a special education class, involvement with a child protection agency, and indications of gender dysphoria such as feeling uncomfortable with their sex assigned at birth or identifying as a member of another gender. Transgender youth were recruited from the Gender Identity Service at CAMH or referred by a private practice clinician. Cisgender participants were recruited from the community via advertisements posted online (e.g., Kijiji, Facebook) and snowball sampling via word of mouth.

Participants were screened for eligibility in person or over the phone. Eligible participants between ages 12 to 15 years of age provided verbal assent and a parent/guardian provided informed, written consent. Participants aged 16 and 17 years provided informed, written consent. The study was conducted in accordance with the Declaration of Helsinki, and the protocol was approved by the Research Ethics Board of the Centre for Addiction and Mental Health (#145–2013). Informed consent was obtained from all subjects involved in the study. At the time of the study session, participants completed a 1-hour imaging session, a brief intelligence assessment and questionnaire package, and a 20-mL blood draw. For most participants, all study procedures were completed on the same day and the order of procedures was allowed to vary. Participants received an honorarium of $20 CAD per hour for their time. Below, measures relevant to the current study are described in detail.

### Measures

#### Age

Age in years was self-reported by participants. To calculate age in months, the date that the study consent form was signed was subtracted from the date of birth and rounded to the nearest hundredth of a decimal.

#### Estradiol

Serum estradiol concentration was derived from a 20 mL serum blood sample collected at the CAMH blood lab. Estradiol concentration was not available for six participants due to opting out of blood collection (*n* = 2 cisgender girls) or had an insufficient sample volume (*n* = 1 cisgender girl; *n* = 3 transgender boys). For eight participants, estradiol concentration was reported as less than 37 pmol/L (*n* = 1 cisgender girl; *n* = 6 cisgender boys; *n* = 1 transgender boy). These eight participants were retained in the analyses using 37.0 pmol/L as the estradiol concentration.

#### Pubertal development scale

Pubertal stage and onset were assessed via self-report on the PDS [40]. The PDS is evaluated based on sex assigned at birth and, thus, consists of five different questions for adolescents assigned male at birth (AMAB) and those assigned female at birth (AFAB) to report on pubertal development. Questions for those AMAB assessed growth spurt, voice change, skin changes, body hair growth, and facial hair growth. For those AFAB, questions assessed growth spurt, breast development, skin changes, body hair growth, and menarche. All participants rated their development on a four-point scale ranging from 1 (has not begun yet) to 4 (seems complete), except on the female menarche question where participants chose from “yes” or “no.” Menarche had occurred for all participants AFAB; thus, this binary item was removed from overall scoring. The remaining responses are averaged for an overall pubertal development score that ranges from 1 (prepubertal) to 4 (post-pubertal). Internal consistency was high on the AMAB subscale (Cronbach’s alpha = 0.94) and moderate on the AFAB subscale (Cronbach’s alpha = 0.76).

#### Gender identity and gender dysphoria

Cisgender and transgender participants’ feelings towards their gender identity were assessed using the self-report Gender Identity/Gender Dysphoria Questionnaire for Adolescents and Adults (GIDYQ-AA [45, 46]). This is a 27-item questionnaire that assesses concurrent gender identity and gender dysphoria in the past 12 months and has been specifically validated for use with adolescents and adults [47]. The GIDYQ-AA is completed based on sex assigned at birth and, thus, the cisgender boys completed the AMAB version, and cisgender girls and transgender boys completed the AFAB version. Each item was rated on a 5-point Likert-type scale from 1 (always) to 5 (never). A mean score was calculated, with lower scores indicating stronger gender dysphoria.

#### Sexual orientation

Sexual orientation was assessed in all participants by self-reported response to the Erotic Response and Orientation Scale (EROS), which queries sexual behavior including sexual attractions in the past 6 months [48]. The 16-item questionnaire asks eight questions about attractions/fantasies to boys/men (i.e., androphilia) and eight questions about attractions/fantasies towards girls/women (i.e., gynephilia) using similar language. Each item is rated on a 5-point scale indicating frequency from 1 (not at all) to 5 (almost every day). Mean androphilia and gynephilia scores were derived for each participant. Higher scores indicate more attractions/fantasies. Internal consistency on both scales was high (Cronbach’s alpha: androphilia = 0.95; gynephilia = 0.95).

The EROS scores were used to generate two sexual attraction variables: strength of attraction (i.e., none to many attractions) and direction (i.e., gynephilia, androphilia) as described in Skorska et al. [39]. A vector reflecting the strength (magnitude) and degree (phase) of sexual attractions was calculated from the individual EROS scores. Androphilia was arbitrarily placed on the *x*-axis and gynephilia on the *y*-axis. The strength of sexual attractions was denoted by the length of the vector, calculated for each participant using the following formula:

$$\sqrt{\left({\left(androphilia\;score\right)}^{2}+{\left(gynephilia\;score\right)}^{2}\right)}$$ (1)

The direction of sexual attractions or degree of androphilia to gynephilia was calculated for each participant using the following formula:

$$\left(arccos\;\left(\frac{androphilia\;score}{magnitude}\right)\right)*\left(\frac{180{}^{\circ}}{\pi }\right)$$ (2)

These values are symmetrically distributed ± 31° around 45° such that a 14° phase indicates exclusive androphilia, 76° indicates exclusive gynephilia, and 45° indicates equal scores on androphilia and gynephilia.

### Neuroimaging

#### Acquisition

Neuroimaging data were collected using a GE MR750 3T MR scanner (General Electric Milwaukee, WI, USA) with an 8-channel head coil (General Electric, 8HR BRAIN, GE Standard 8-channel head coil) at CAMH. Acquisition of DTI and T2-weighted data were obtained as part of the 1-hour scanning session. Diffusion tensor images were acquired using a 2D spin echo pulse imaging sequence with axial slices; 2 mm isotropic resolution; b = 1000 mm^2^/s; 60 diffusion weighted and 5 non-diffusion weighted images. T2-weighted images were acquired with an FSE-XL pulse sequence in the axial plane; flip angle = 125°, field of view = 22 cm, 256 mm x 256 mm matrix, 3 mm thick slices.

T1 mapping was used to further characterize WM tissue. T1 relaxation time maps depict the recovery of longitudinal magnetization. This is known to be sensitive to the microstructure surrounding the water molecules, which are the source of MRI signal. Thus, differences in T1 values reflect differences in tissue microstructure. T1 maps with B1 correction were calculated from four acquisitions using sagittal spoiled-gradient echo (SPGR) pulse sequences. Two of these images, referred to as Flip14 and Flip3, were acquired by fast-SPGR scans (repetition time = 10.6 ms, echo time = ~4.4 ms, acquisition time = 2:59 min each) with 1 mm isotropic resolution (field of view = 25.6 cm, 160 slices) at 14° and 3° flip angles, respectively. The Flip14 and Flip3 images were used for T1 mapping calculations using the variable flip angle method with a calibration step [49]. To account for B1 inhomogeneities, B1 maps were computed using an extrapolation to signal null per the method of slopes [50] from two SPGR scans (repetition time = 50–60 ms, echo time = 5 ms, acquisition time = 2:24 min each) with low 4 mm isotropic resolution (field of view = 25.6 cm, 40 slices) and two flip angles (130° and 150°).

#### Processing

All neuroimaging data were processed using FMRIB Software Library (FSL, v 6.0 [51]).

#### Diffusion maps

Diffusion-weighted images were first corrected for susceptibility distortion due to the EPI acquisition. The mean non-diffusion weighted (b = 0) image was non-linearly registered to a non-EPI based T2-weighted image collected using a fast-spin-echo (FSE) sequence. This was accomplished using FNIRT [52, 53]. The *warpres* parameter was used to constrain the transformation to the y-plane only to account for posterior-anterior distortion of EPI images along the phase-encoding direction. Next, the images were corrected for eddy current distortions using FSL’s eddy algorithm [54]. The distortion-corrected diffusion-weighted data were then processed using FSL’s DTIFIT to fit each voxel with a diffusion tensor model. Maps were calculated for FA, MD, AD, and RD.

The TBSS pipeline with default parameters [55] was used to register individual FA maps to MNI standard space. A threshold of 0.2 was applied to the mean FA skeleton to reduce partial volume effects. A WM skeleton was created from the mean FA image of the sample. The non-FA brain maps (AD, RD, MD) were then transformed to MNI standard space using the transformations determined by the TBSS pipeline.

#### T1 maps

The four SPGR images used to create the T1 maps were reoriented/coregistered into the space defined as halfway between the Flip3 and Flip14 images using the “halfway flirt” command from FSL’s SIENA pipeline. The Flip3 image was brain-extracted using FSL’s brain extraction tool (BET) and the resulting mask was used to extract the brain of the Flip14 image. T1 maps were calculated and calibrated as described previously by Chavez [49]. Further processing of the T1 maps included linear registration to the FSE T2-weighted image using FSL’s FLIRT with a mutual information cost function. Then, the T1 maps were transformed to MNI standard space using the non-FA TBSS pipeline. The T1 maps were smoothed with a median filter using a kernel of 3 voxels before the WM skeleton was applied.

#### White matter skeleton

For the partial least squares (PLS) analyses only, the sample mean WM skeleton generated with TBSS was dilated to include more WM tissue in the analyses. Using FSL’s FAST, the WM was segmented from the average of all subjects’ T1-weighted images and used to mask the dilated WM skeleton to ensure the dilated skeleton stayed within WM tissue across subjects. This study sample-specific WM skeleton (S1 Fig) was then applied to the diffusion indices and to the smoothed T1 maps. Finally, before the multivariate analyses, the AD map was scaled by 10^5^ to approximate the range seen for T1 relaxation times.

### Statistical analysis

#### Demographics and psychosexual variables

Group differences in age, pubertal development (i.e., PDS scores), estradiol concentration, gender dysphoria (i.e., GIDYQ-AA scores), and strength and direction of sexual attraction (derived from EROS scores) were assessed by one-way analyses of variance (ANOVA) using SPSS version 28 (IMB Corp., 2021). Post hoc pairwise comparisons were performed using Fisher’s least significant difference (LSD) when omnibus ANOVA effects were statistically significant. A conventional alpha level of *p* < 0.05 was used to indicate statistical significance.

#### TBSS

A one-way ANOVA assessed group differences for each diffusion map (i.e., FA, MD, AD, RD). “Randomise” [56] was used to apply whole-brain, voxel-wise statistics with 5000 permutations and threshold free cluster enhancement (TFCE) to identify group differences for each metric independently. FSL’s cluster tool was used to identify significant clusters (i.e., *p* < 0.05 and *k* > 100 voxels).

#### Multivariate analyses

The TBSS analyses identified group differences in AD only. Therefore, to broaden our interpretation of WM tissue composition, a complementary measure of microstructure, T1 relaxation time, was included in the multivariate analyses. T1 is a myelin-sensitive parameter [27]. Two PLS analyses were performed using methods outlined by McIntosh and Lobaugh [57] and conducted using PLS software (v. 6.1311050) with MATLAB (2014a, version 8.3.0.532). This multivariate approach is a robust analytical tool for identifying distributed patterns in structural brain data [58]. The first PLS analysis was designed to identify similarities and differences in how the WM microstructure metrics were modulated by group membership. Each metric was separately mean-centered across all subjects to minimize the detection of scalar differences between AD and T1. PLS uses singular value decomposition on mean-centred data to identify the dominant relationships, creating latent variables (LVs) with three components: contrasts reflecting group and/or metric differences, brain saliences (i.e., a measure of the strength of each voxel’s contribution to the observed contrast), and a measure of relationship strength [57]. The dot-product of the brain saliences with a participant’s data produces a brain score, indicating how strongly each individual participant reflects the contrast identified by the latent variable. The relationship strength is used to evaluate the statistical significance of the model. Permutation testing assessed the number of times the strength of the permuted LVs exceeded the observed strength and, thus, determined the probability of the model. The stability of the voxel contributions to the contrast was assessed with bootstrap resampling to estimate the standard error of the brain saliences. The ratio of the brain salience to its standard error (bootstrap ratio) approximates a z-score and was used to assess the reliability and stability of the salience. A threshold of ±2.5 was applied to ensure only stable saliences were included.

The first PLS analysis assessed the relationship of group membership (i.e., cisgender girls, cisgender boys, transgender boys) with WM microstructure using AD and T1. The second PLS analysis assessed the relationship of pubertal development metrics with the two WM microstructure metrics. Specifically, the analysis examined the correlation of AD and T1 data with serum estradiol concentration (pmol/L), age (months), average PDS score, and strength and direction of sexual attractions. Both analyses used 5000 permutations and bootstrap samples. The regional labels for stable voxels were derived using the John Hopkins University (JHU) DTI-based white-matter atlases [59].

## Results

### Demographic and psychosexual analyses

Descriptive statistics for each demographic and psychosexual variable are shown in Table 1 by group (correlations between variables are reported in S2 Table). The three groups did not significantly differ in age (*F*(2,45) = 0.68, *p* = 0.51, *η*^2^ = 0.03) or pubertal development (PDS: *F*(2,45) = 1.37, *p* = 0.26, *η*^2^ = 0.06), but estradiol concentration was significantly lower in cisgender boys compared with cisgender girls and transgender boys (*F*(2,39) = 5.95, *p* = 0.06, *η*^2^ = 0.24; all pairwise *p <* 0.02). Mean estradiol concentration did not differ between cisgender girls and transgender boys (*p* = 0.48).

**Table 1:** Demographic and developmental variables of study sample by group.

Variable (mean, SD)	Cisgender Girls	Cisgender Boys	Transgender Boys	*F* (*df*)	*p* (*η*^2^)
***n***	17	14	15		
**Age (years)**	15.47 (1.62)	14.79 (2.04)	15.60 (1.24)	0.68 (2,45)	0.51 (0.03)
**PDS**	3.13 (0.53)	2.86 (0.78)	3.23 (0.58)	1.37 (2,45)	0.26 (0.06)
**Estradiol (pmol/L)**	369.57 (350.28)^a^	73.36 (44.48)	302.83 (205.00)^a^	5.95 (2,39)	0.06 (0.24)
**GIDYQ-AA**	4.90 (0.15)	4.91 (0.12)	2.17 (0.33)	777.04 (2,45)	< 0.001 (0.97)
**Strength of Sexual Attraction**	2.83 (0.76)	3.25 (1.28)	2.98 (1.34)	0.53 (2,43)	0.59 (0.24)
**Direction of Sexual Attraction**	30.94 (13.18)	64.60 (9.29)	46.99 (15.56)	25.67 (2,43)	< 0.001 (0.54)

^a^sample size for estradiol was reduced due to individual limitations during blood collection (cisgender girls: *n* = 14; transgender boys: *n* = 12). GIDYQ-AA = Gender Identity/Gender Dysphoria Questionnaire for Adolescents and Adults.

Scores on the GIDYQ-AA significantly differed by group (*F*(2,45) = 777.04, *p* < 0.001, η^2^ = 0.97), whereby transgender boys had significantly lower scores than cisgender girls and cisgender boys (all pairwise *p* < 0.001) who did not differ from each other (*p* = 0.89). A GIDYQ-AA score ≤ 3.0 is used as the threshold for potential gender dysphoria diagnosis [60]. The lowest score on the GIDYQ-AA in the cisgender participants was 4.48, indicating no potential gender dysphoria diagnoses among the cisgender groups, and the highest score in the transgender participants was 3.04.

**Fig 1:**
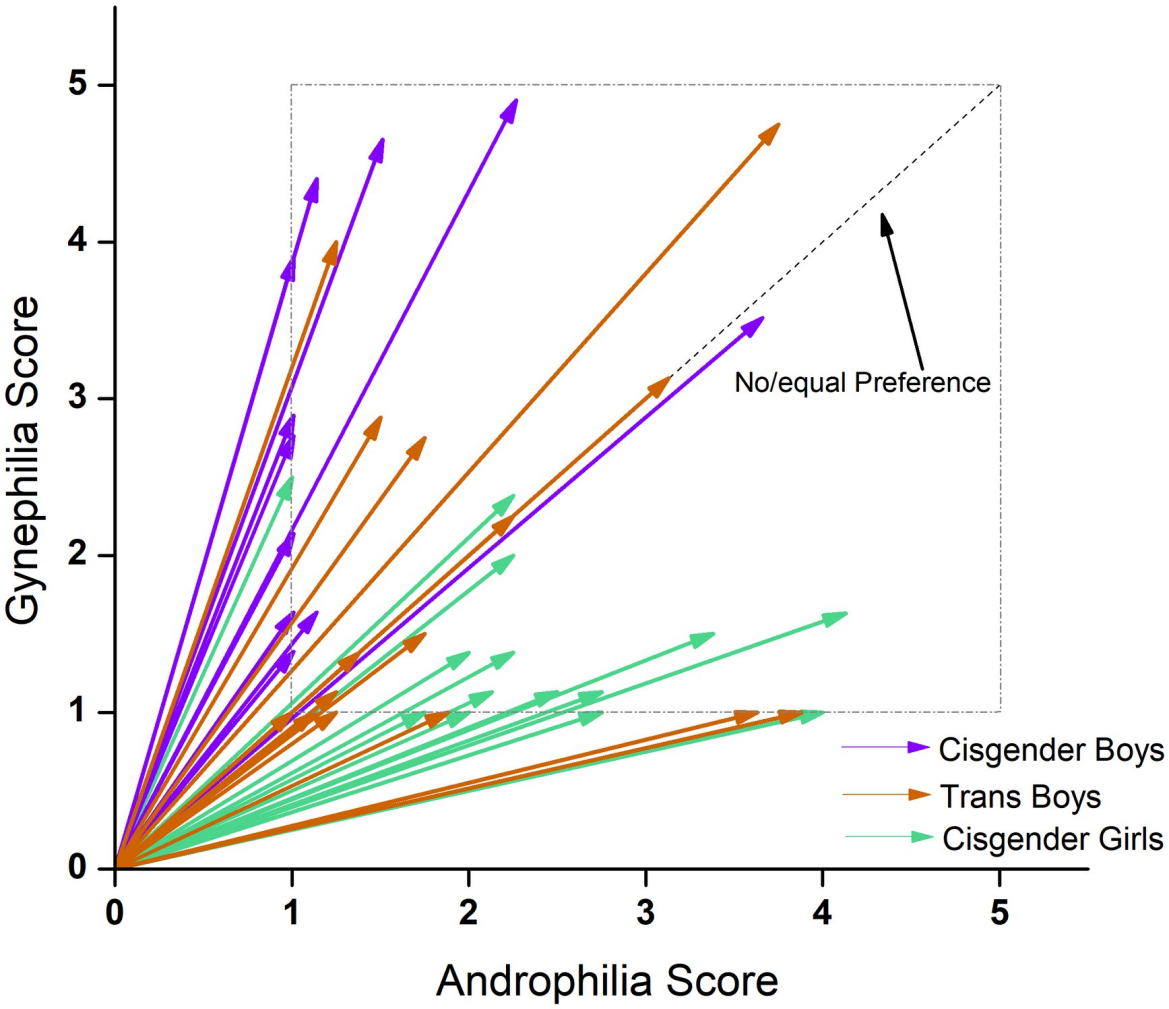
Direction and strength of sexual attractions derived from EROS. Direction of sexual attraction is calculated from the degree of gynephilia-androphilia. Dashed centre line at 45° represents no or equal preference. Solid arrows depict degree of gynephilia and androphilia with a maximum for this sample of ± 31°. The strength of sexual attraction is determined by the length of the vector, independent of the direction of the vector. Cisgender boys (purple) were predominantly gynephilic, transgender boys (orange) expressed a range of sexual attractions, and cisgender girls (green) were predominantly androphilic.

Also, strength of sexual attractions did not differ significantly across groups (*F*(2,43) = 0.53, *p* = 0.59, *η*^2^ = 0.24), but direction of sexual attractions showed a significant effect of group (*F*(2,43) = 25.67, *p* < 0.001, *η*^2^ = 0.54; Fig 1). Cisgender boys were more gynephilic than cisgender girls (mean difference (MD) = 33.66, standard error (SE) = 4.70, *p* < 0.01) and transgender boys (MD = 17.61, SE = 4.84, *p* = 0.002), and cisgender girls were more androphilic than transgender boys (MD = -16.1, SE = 4.61, *p* = 0.003). The transgender boys had a range of sexual attractions, including a greater proportion of participants in the ‘no/equal preference’ range.

### TBSS analyses

**Fig 2:**
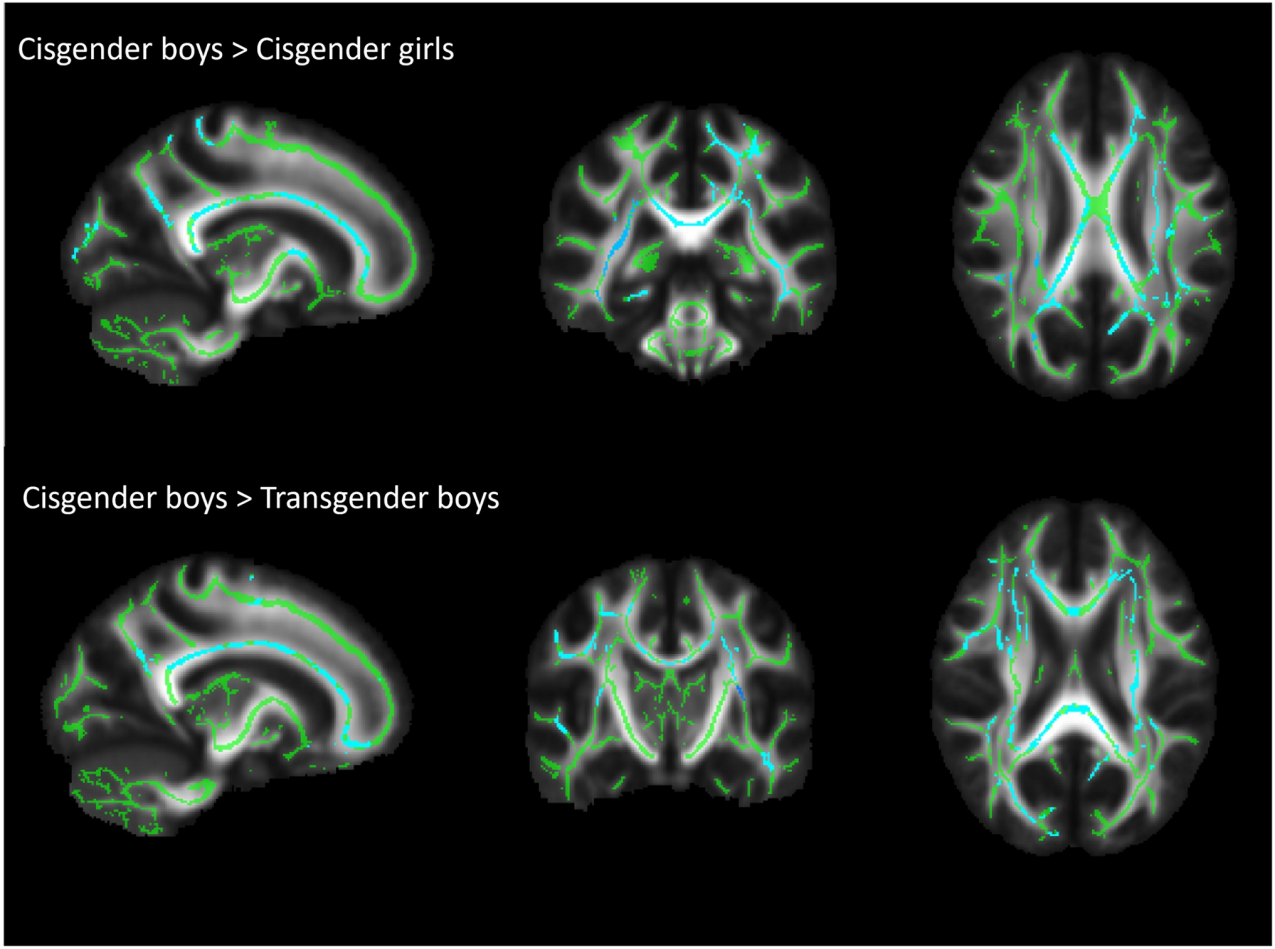
Significant axial diffusivity clusters from TBSS analysis. TBSS analyses reveal greater AD in cisgender boys compared with cisgender girls and transgender boys. Significant clusters (*p* < 0.05, *k* > 100; light blue) are overlaid on the sample mean FA skeleton (green). The underlying map is the sample mean FA map. Anatomical left is right.

The one-way ANOVAs identified significant group differences for AD only (*F*-test; *p* = 0.021). Post-hoc two-group comparisons revealed that after TFCE, AD was higher in cisgender boys compared with transgender boys (*p* = 0.004) and cisgender girls (*p* = 0.008), who did not differ from each other (*p* = 0.59). Significant clusters remaining after TFCE were widespread in most major WM tracts (Fig 2; Table 2): bilaterally in the forceps major and minor, corpus callosum (genu, splenium, and body), inferior frontal occipital fasciculi (IFOF), and corticospinal tract (CST); the right inferior longitudinal fasciculus (ILF); and the left anterior thalamic radiation and superior longitudinal fasciculus (SLF). No significant group differences were found for FA, MD, or RD after TFCE.

**Table 2:** Significant clusters revealed for AD using FSL’s cluster tool.

Cluster	*k* (voxel number)	*p*-value
Cisgender boys > cisgender girls		
1	15115	0.002
2	2192	0.024
Cisgender boys > transgender boys		
1	20951	0.002

Additionally, ad-hoc analyses were performed to consider the influence of age. Using an ANCOVA design with 5000 permutations and age as a covariate, it was found that age was negatively associated with AD, RD, and MD, but not FA. Pairwise comparison results between groups were unchanged after covarying for age. In other words, AD remained significantly higher in cisgender boys compared with cisgender girls (*p* = 0.005) and transgender boys (*p* = 0.009), and there were no significant pairwise differences found with RD or MD. Further detail of the ad-hoc analyses can be found in the SI.

### PLS analyses

The group PLS concurrently assessing both AD and T1 relaxation time did not reveal any significant group differences. The first LV, explaining 29.62% of the cross-block covariance, approached significance (*p* = 0.081). Although this LV did not show a significant effect, descriptively the pattern associated with this LV, which distinguished cisgender boys from cisgender girls and transgender boys, was noteworthy in that AD and T1 had similar patterns within each group (S3 Fig). Overall, AD was higher and T1 was longer in cisgender boys relative to the other two groups. Stable voxels were found primarily in major WM tracts (e.g., cingulum bundle, corpus callosum, optic radiations, and SLF; see S3 Fig).

**Fig 3:**
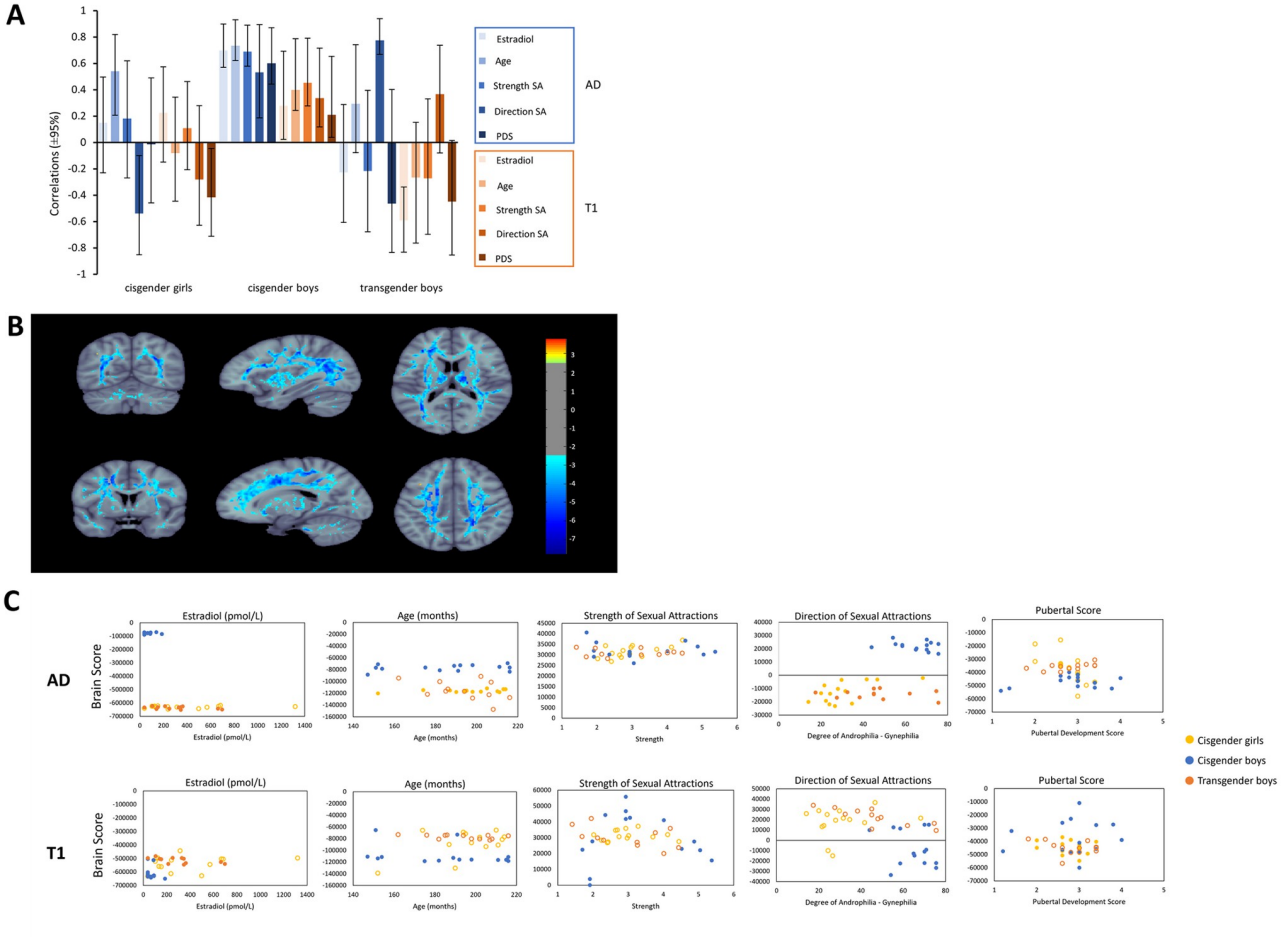
Proxies of pubertal development PLS LV1. **A.** Proxies of Pubertal Development PLS LV1 correlations. Error bars indicate bootstrap-estimated ±95% confidence intervals. Correlations with confidence intervals that do not overlap with zero indicate a stable contribution to the PLS LV1. Shades of blue and orange correspond to correlations between pubertal development variables and AD and T1 relaxation time brain scores, respectively. SA = sexual attractions; PDS = pubertal development score. **B.** Visualization of ±2.5 bootstrap ratio from LV1 of developmental PLS. Analysis indicated widespread AD and T1 correlations with the five developmental variables. Anatomical left is left for coronal and axial view; in sagittal view left is anterior, right is posterior. **C.** Scatterplots illustrate the brain score-behavior correlations for each developmental measure with AD and T1 brain metrics for each group. Stable correlations are indicated by filled markers and correspond with the confidence intervals in A. The strength of each correlation is illustrated in A and listed in S4 Table.

In the pubertal development PLS, two significant LVs were identified. The effects identified on both LVs were dominated by AD and T1 correlations with the developmental measures in the cisgender boys. LV1 (*p* < 0.001) explained 26.39% of the model’s covariance and indicated the whole brain-behavior correlations were generally negative, similar in strength, and common across all five behavioral measures for both AD and T1 in cisgender boys. This LV also included some stable correlations in AD and T1 in cisgender girls (i.e., AD: negatively with age; positively with direction of sexual attractions; T1: positively with PDS) and transgender boys (i.e., AD: negatively with direction of sexual attractions; T1: positively with estradiol), some of which matched the direction of correlations in cisgender boys (Fig 3A). The voxels contributing to this pattern were found throughout the WM skeleton (Fig 3B).

The second significant LV (LV2, *p* = 0.001) explained 10.97% of the model’s covariance and described a pattern of correlations that differentiated AD-development correlations from T1 relaxation-development correlations, again primarily in cisgender boys. In this case, the LV indicated that T1 relaxation showed consistently weaker correlations than AD for all developmental measures in the cisgender boys. Only PDS-T1 correlations were stable in cisgender girls and transgender boys, and these were in the opposite direction of those in cisgender boys. The stable positive and negative voxel saliences were scattered throughout the skeleton and were not localized to any one region. Many of the strong positive and strong negative AD-developmental correlations were found at the edges of the dilated WM tract, and may have reflected partial volume effects, therefore, this LV was not interpreted further (see S4 Fig).

## Discussion

The present study investigated sex/gender and pubertal development differentiation in WM microstructure in cisgender and transgender adolescents. We sought to overcome some limitations of previous DTI research such as restricting measurement to the center of the WM tract (i.e., TBSS; [55]) and employing mass-univariate statistics on diffusion metrics independently. To do so, the present study conducted PLS analyses that used a dilated WM skeleton to capture more WM tissue in the analysis, included multiparametric mapping to improve the inferences made about tissue properties, and applied multivariate statistics to examine associations with developmental and sexual attraction variables. The findings indicated that WM microstructure, as measured by AD and T1, was differentiated by group and associated with pubertal development and sexual attraction.

The TBSS analysis indicated a group difference in AD whereby cisgender boys had higher AD than transgender boys and cisgender girls bilaterally in the corpus callosum, forceps major and minor, IFOF, and CST, the right ILF, and the left thalamus radiation and SLF. This pattern aligns with the findings of other adolescent studies that analyzed AD using TBSS [23, 24, 28]. These studies also reported sex-related differences in FA, MD, and RD among cisgender adolescents, though not always in the same WM regions, whereas widespread differences in the three indices were not present in the current analysis. Changes to AD and RD often result in changes to the so-called summary measures, FA and MD. FA and MD include the primary, secondary, and tertiary diffusivities of a voxel and are commonly reported to simplify the characteristics of water diffusion [20]. Therefore, FA and MD are less specific and may have more noise relative to AD, which only includes the primary diffusivity [20]. The lack of difference in FA and MD, relative to the significant difference in AD in the present study, could be due to increased noise.

The subsequent group PLS showed a parallel, but non-significant pattern whereby higher AD combined with longer T1 differentiated cisgender boys from the other two groups. Given that AD and T1 are seldom investigated together, the current group PLS findings require cautious interpretation. However, despite the pattern being non-significant, the pattern of AD and T1 is an important avenue to explore in future research to aid our interpretation of WM tissue characteristics. First, the interpretation of AD is complex. Because WM neurodevelopment is characterized by increased axonal straightening, myelination, and tissue density [10], high AD could be considered a potential indicator of axonal straightening and organization and, therefore, related to greater WM maturation [61, 62]. Alternatively, increases to WM tissue density, as determined by the number of axons, axonal calibre, and the thickness of the myelin sheath, is found to lead to the age-related increase in WM volume [14]. Therefore, high AD may also be indicative of less axonal density [19, 28]. Second, T1 relaxation time can inform the degree of myelination [27]. Given that T1 relaxation time is sensitive to macromolecules in neural tissue, longer T1 values in WM may be interpreted as reflecting less myelination [27]. Thus, in addition to the TBSS analysis, we can speculate that the parallel pattern shared between AD and T1 may suggest that the axons are less densely packed and myelinated in the cisgender boys compared with the other two groups. Previous studies of age-matched cisgender adolescents have demonstrated greater WM microstructure maturation in adolescents AFAB compared with those AMAB [22–24, 28]. The data may also reflect a tentative sex/gender difference in WM maturation whereby cisgender boys experience a decrease in axonal density that is not found in adolescents AFAB (i.e., [13, 63]).

Our study is the first to assess AD alongside T1 in transgender adolescents, but recently, van Heesewijk et al. [7] reported on FA in transgender children, as well as adolescents during puberty suppression. Including both transgender boys and girls, van Heesewijk et al. [7] reported lower FA in transgender adolescents compared with age-matched cisgender adolescents. Individual univariate analyses of major WM tracts revealed that adolescents AMAB had higher FA in the right SLF and left CST compared with adolescents AFAB, regardless of gender identity [7], potentially indicating that cisgender boys and transgender girls have greater directionality in their WM microstructure than the other groups. Here, we did not find any significant relationships with FA, but the TBSS analysis did find higher AD, which could reflect less axonal density [19] or more axonal organization [62], in cisgender boys compared with both cisgender girls and transgender boys. Importantly, the transgender adolescents in van Heesewijk et al. [7] had received at least two months of puberty suppression GAHT, whereas the transgender boys in the present study were naïve to any GAHT. The inclusion of exogenous hormone therapy confounds any comparison with the present study; however, both studies highlight the complexity of characterizing patterns of WM microstructure development across sex/gender.

When AD and T1 were examined in relation to developmental measures, we found that both indices of WM microstructure were associated with all five chosen metrics of pubertal development and sexual attraction in cisgender boys. Notably, in LV1, all the variables were negatively correlated with AD and T1 in cisgender boys, potentially indicating that as pubertal development progressed, WM microstructure matured. This relationship was found in extensive regions in the IFOF, ILF, and SLF—three WM tracts that have been reported to reach complete maturity by mid-puberty in cisgender boys and girls [6, 22, 64], but see [10]. WM microstructure metrics did not correlate with as many measures in the participants AFAB (i.e., cisgender girls, transgender boys). This discrepancy could be due to sex/gender differences in development (i.e., protracted neurodevelopment in cisgender boys [24]. The neurodevelopmental differences could also reflect a difference in developmental timeline, such that cisgender boys reach peak neurodevelopment (i.e., high tissue organization, low diffusion [10]) at different stages of pubertal development than those AFAB, and thus, correlations in the groups AFAB are less pronounced in comparison.

The concurrent investigation of AD and T1 with pubertal development metrics is novel and yet challenging to interpret in the context of previous literature. We now delve into some of the findings that go beyond the primary finding of AD/T1 effects in cisgender boys. Direction of sexual attraction correlated with AD negatively in cisgender and transgender boys, and positively in cisgender girls. The majority of cisgender boys reported some degree of gynephilic attraction, whereas a range of gynephilic and androphilic attractions were reported by transgender boys. The findings potentially suggest that the combination of gynephilic sexual attraction and a masculine gender identity are associated with more axonal packing or density. Gynephilic attraction was also related to shorter T1 in cisgender boys, indicating more myelination. These WM findings could extend to previous gray matter morphometric/structural findings from a largely overlapping cohort [39]. It was found that greater gynephilic attractions were related to shorter T1 in several cortical regions across the brain in cisgender and transgender boys [39]. Together, the studies potentially describe a neural phenotype for gynephilia in masculine-identifying individuals.

AD was positively correlated with direction of sexual attraction in cisgender girls, who mostly reported some degree of androphilic attraction. This relationship opposes the negative correlation in cisgender and transgender boys and may suggest that greater axonal density is associated with a higher degree of androphilia specifically in feminine-identifying individuals. Although these inferences would benefit from the inclusion of more sexual diversity (e.g., another feminine gender group), the relationships do demonstrate that sexual attraction metrics should be considered in future WM microstructure research.

Inclusion of estradiol concentration in the present study also provided a preliminary assessment of the impact of the pubertal hormone environment on WM microstructure and aspects of pubertal development. Estradiol and T1 were related in opposite directions in cisgender and transgender boys such that it can be interpreted that lower estradiol was related to fewer macromolecules (e.g., less myelin) in cisgender boys, but more macromolecules (e.g., more myelin) in transgender boys. In a post-hoc PLS analysis of AD and T1 with estradiol alone using 5000 permutations and bootstrap resampling, this relationship was preserved (see S5 Fig). Cisgender boys had significantly lower overall estradiol concentration than cisgender girls and transgender boys, who did not differ. The estrogenic-T1 relationship in transgender boys may indicate that estrogen interacts with WM differently in this group compared with cisgender girls and boys. Given that transgender boys and cisgender girls are both assigned female at birth and have comparable estrogenic concentrations, but have different gender identities, the discrepant estrogenic relationship may indicate that estrogen interacts with WM differently by gender identity in individuals assigned female at birth, such that low estradiol promotes, or high estradiol reduces, myelination in transgender boys.

This estrogenic relationship could have clinical implications for transgender boys who begin puberty suppression GAHT. Puberty suppression prevents undesired secondary sex characteristics (e.g., breasts) from developing by over-saturating gonadotropin releasing hormone receptors and blocking the release of estradiol and testosterone [65]. In other words, estradiol concentrations are decreased in transgender boys using puberty suppression GAHT. Although the present study can only offer insight to endogenous hormone associations and not the use of puberty suppression GAHT, the findings describe an inverse relationship between estrogen and myelin that may be myelin-promoting and that is potentially unique to transgender boys. Healthcare providers face some challenges in providing GAHT, including puberty suppression, to adolescents [5, 66] and literature on the impact puberty suppression has on neural structure, function, and behavior in transgender adolescents is currently limited, but is demonstrated to positively impact mental health [67]. Therefore, it is worth further exploring estrogenic relationships with measures that inform about myelin in transgender adolescents given myelin’s role in signal conduction [11].

It is important to note that the reported differences in WM microstructure in this study are not characterized as aberrant or clinically relevant differences. That is to say, the study design prevents causal interpretation of the reported differences with respect to group membership (i.e., cisgender boy, cisgender girl, transgender boy) or other demographic information (i.e., diagnosis of gender dysphoria; estradiol concentration). The present findings cannot be used to infer hierarchical phenotypes on the bases of sex, gender, or sexual attraction.

### Limitations

The present findings are limited by small sample sizes. PLS, a multivariate analysis that utilizes the covariance across variables, helps highlight the relationships in our data while circumventing some of the strict statistical corrections common to mass-univariate methods, which are often emphasized in small datasets. Although some PLS methods have been demonstrated to be limited by small sample size (e.g., [68]), the present studies used correlative PLS, which are not as limited by sample size constraints [58]. The inclusion of estradiol in the developmental PLS analysis further limited the sample size due to challenges in blood collection. Collecting estradiol concentration from a single timepoint also limits the interpretation of these findings. Biobehavioral research recommends capturing hormonal fluctuations with multiple samples, but collection of an absolute level can be sufficient when sampling blood [69].

The PLS analyses were performed on a dilated WM skeleton that was created from the TBSS-generated mean sample skeleton. The purpose of the dilation was to increase the data available for multivariate analysis by expanding the skeleton to include WM tissue beyond the centre of the fiber bundles [55]. This method may have inadvertently introduced the risk of partial volume effects, such that non-WM tissue may have been included in analysis. However, to assure this risk was minimized, the creation of the dilated WM skeleton included a WM segmentation step using FSL’s FAST algorithm to ensure that dilation of the skeleton was still limited to WM tissue only.

The investigation of WM microstructure may have benefitted from the additional inclusion of neurite orientation dispersion and density imaging (NODDI) parameters. These parameters have been more recently developed to improve assessment of water signals in multi-shell diffusion MRI data [70]. The present data were acquired using single-shell diffusion, the common acquisition at the time of data collection, which poses some challenges to applying NODDI parameters [70]. Future investigations or replication of this work should consider deriving these metrics for greater characterization of WM tissue.

The interpretations were further limited in their generalizability to cisgender and transgender adolescents due to the homogeneity of the sample. For instance, the direction of sexual attraction findings should be replicated in a more sexually diverse cisgender sample to better disentangle the influence of sex/gender from sexual attraction on WM microstructure development. Furthermore, the requirement of a diagnosis of gender dysphoria for participation in neuroscientific studies from transgender adolescents limits the study’s scope of gender diversity to healthcare and institutional settings only, preventing the findings from applying more generally to transgender and nonbinary individuals [71]. Additionally, the present study was unable to assess any direct associations between WM microstructure and behavior. The cognitive and behavioral implication of WM “integrity” in typical brain development is inconclusive for reasons such as the “architectural paradigm,” which describes the fallacy of classifying voxels with identical tissue composition but different fibre organization as differentially “integral” based on anisotropy alone [25].

## Conclusions

The present study contributes to a nascent literature of transgender adolescent neuroimaging. More specifically, for the first time, the study assessed FA, MD, RD, AD, and T1 in WM microstructure of hormone-naïve transgender adolescents. Group comparisons indicated that AD was aligned with T1 in all groups and cisgender boys had higher and longer values, respectively, compared with transgender boys and cisgender girls. The five developmental metrics were strongly and negatively related to AD and T1 in cisgender boys. Weaker and sometimes inverse associations were found in cisgender girls and transgender boys. More specifically, AD related to direction of sexual attraction (i.e., gynephilia, androphilia) in all groups, and T1 related to estradiol inversely in cisgender boys compared with transgender boys. Therefore, the present study unveiled potentially unique brain patterns relating to WM microstructure and pubertal development in cisgender boys, transgender boys, and cisgender girls with respect to developmental characteristics and WM tissue composition. The unique brain patterns challenge the popular notion of gender-typical or sex-typical neural phenotypes, as well as an intermediate transgender phenotype. Overall, these findings highlight the importance of considering developmental factors when investigating the subtleties of neural structure associated with variations in sex/gender, gender identity, and sexual attraction in adolescence.

## Supporting information

S1 Fig TBSS-generated mean FA skeleton. Sample mean FA skeleton (blue) overlaid on dilated WM skeleton (red) used in statistical analyses. Both skeletons are projected on the sample mean FA map.
(TIF)

S2 Fig PLS analysis of AD, RD, MD, FA, and T1. A group PLS analysis across all metrics was non-significant (all LV with *p >* 0.2). **Top panel:** Brain scores across metrics and groups for LV1. Error bars indicate ±95% confidence intervals. 1 = cisgender girls; 2 = cisgender boys; 3 = transgender boys. **Bottom panel:** LV1 stable regions with bootstrap ratio threshold of ±2.5.
(TIF)

S3 Fig Group PLS non-significant LV1. **A.** Group PLS LV1 brain scores showing non-significant (*p* = 0.081) shared covariance in AD and T1. Error bars indicate ± 95% confidence intervals. AD was higher and T1 relaxation was longer in cisgender boys than cisgender girls and transgender boys. **B.** Stable regions of the group PLS. Bootstrap ratio is thresholded at ±2.5 as indicated by gradient scale on the right. Anatomical left is left for coronal and axial view; in sagittal view left is anterior, right is posterior. The brain pattern was found most stable bilaterally in the IFOF, ILF, splenium of the corpus callosum, left SLF, anterior corona radiata, forceps minor, and forceps major.
(TIF)

S4 Fig Proxies of developmental PLS LV2. **A.** Developmental PLS LV2 (*p* = 0.001) accounting for 10.97% of model covariance. Error bars indicate ±95% confidence intervals. Shades of blue and orange correspond to correlations between pubertal development variables and AD and T1 relaxation time brain scores, respectively. **B.** Regions with stable brain salience of the developmental PLS. Bootstrap ratio is thresholded at ±2.5 as indicated by gradient scale on the right. Anatomical left is left.
(TIF)

S5 Fig Behavior PLS analysis of AD and T1 with estradiol alone. **A.** LV1 (*p* = 0.033) accounted for 36.46% of the model covariance. **B.** Correlation maps of each group depicting differences in strength and direction of T1-estradiol correlation without thresholding the bootstrap ratio. Anatomical left is left.
(TIF)

S1 Table Frequency of self-reported ethnicities of the participants. (PDF)

S2 Table Correlations of demographic and developmental variables with age and estradiol levels across all participants and within group. (PDF)

S3 Table AD, RD, and MD are negatively associated with age in TBSS analysis. (PDF)

S4 Table Correlation between each of AD and T1 with brain scores as a result of PLS. (PDF)
