# Supplementary figures and images for: White matter microstructure in transmasculine and cisgender adolescents: A multiparametric and multivariate study
Source: PLoS One. 2024 Mar 12;19(3):e0300139. doi: 10.1371/journal.pone.0300139 (PMC10931471; doi:10.1371/journal.pone.0300139)

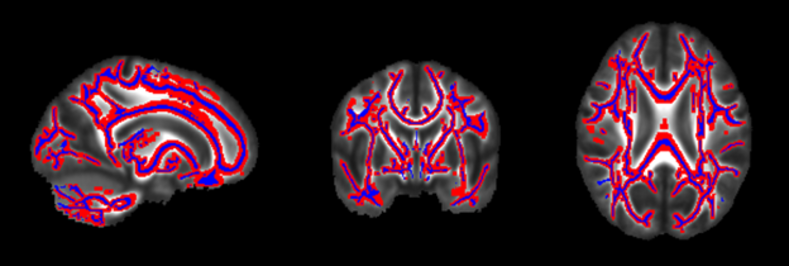

Supplement: S1 Fig — Sample mean FA skeleton (blue) overlaid on dilated WM skeleton (red) used in statistical analyses. Both skeletons are projected on the sample mean FA map. (TIF) [file pone.0300139.s001.tif]

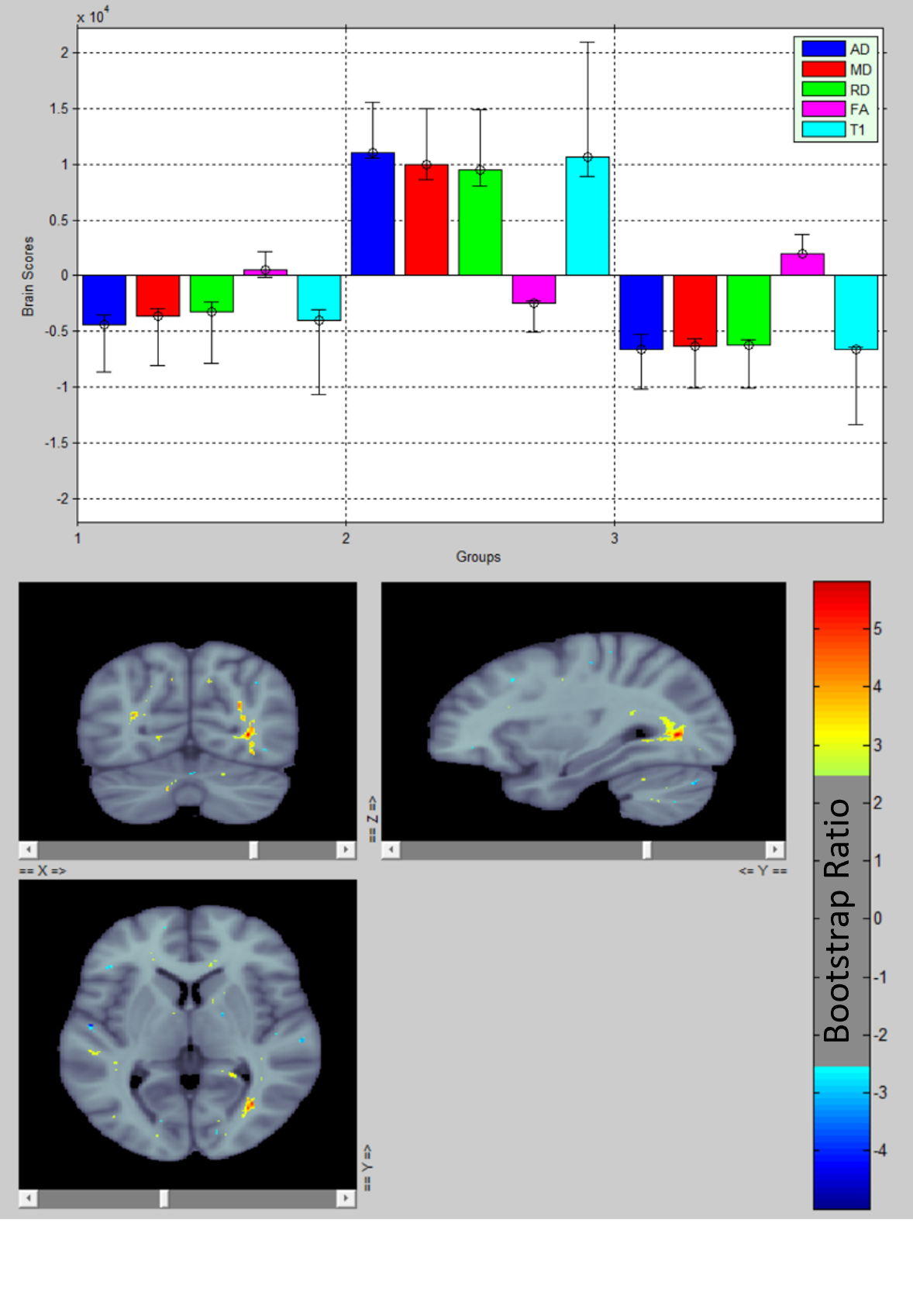

Supplement: S2 Fig — A group PLS analysis across all metrics was non-significant (all LV with p > 0.2). Top panel: Brain scores across metrics and groups for LV1. Error bars indicate ±95% confidence intervals. 1 = cisgender girls; 2 = cisgender boys; 3 = transgender boys. Bottom panel: LV1 stable regions with bootstrap ratio threshold of ±2.5. (TIF) [file pone.0300139.s002.tif]

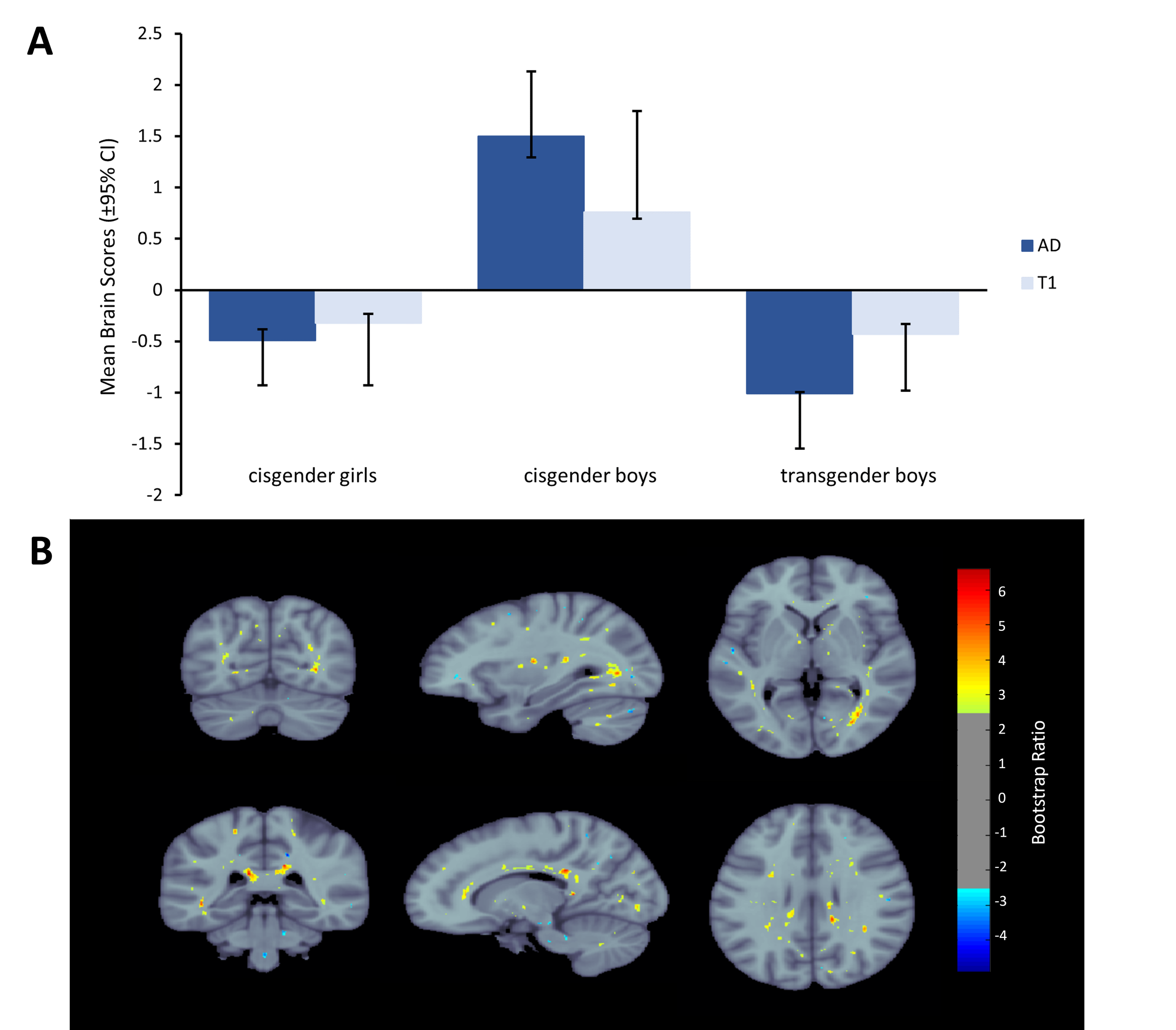

Supplement: S3 Fig — A. Group PLS LV1 brain scores showing non-significant (p = 0.081) shared covariance in AD and T1. Error bars indicate ± 95% confidence intervals. AD was higher and T1 relaxation was longer in cisgender boys than cisgender girls and transgender boys. B. Stable regions of the group PLS. Bootstrap ratio is thresholded at ±2.5 as indicated by gradient scale on the right. Anatomical left is left for coronal and axial view; in sagittal view left is anterior, right is posterior. The brain pattern was found most stable bilaterally in the IFOF, ILF, splenium of the corpus callosum, left SLF, anterior corona radiata, forceps minor, and forceps major. (TIF) [file pone.0300139.s003.tif]

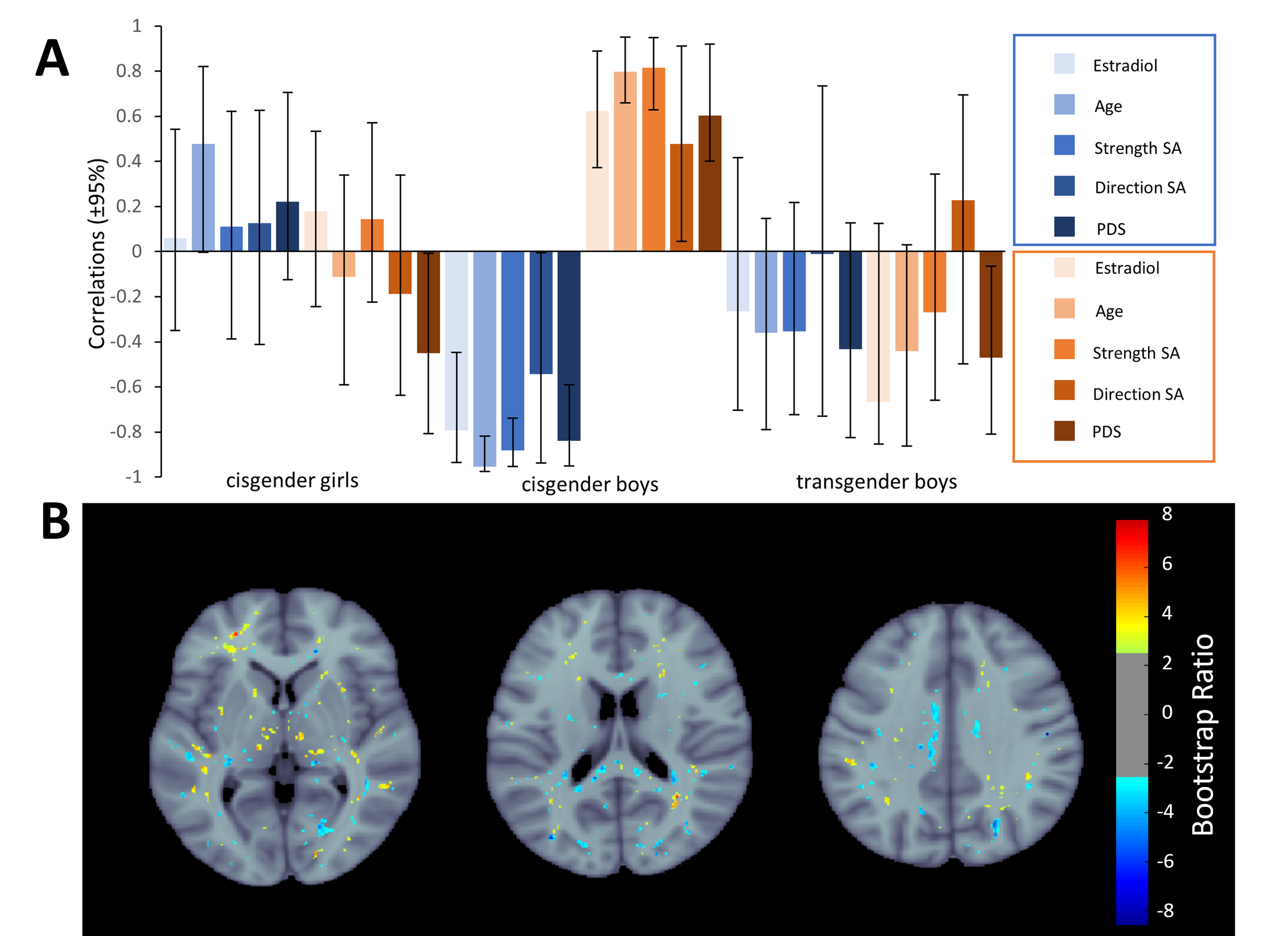

Supplement: S4 Fig — A. Developmental PLS LV2 (p = 0.001) accounting for 10.97% of model covariance. Error bars indicate ±95% confidence intervals. Shades of blue and orange correspond to correlations between pubertal development variables and AD and T1 relaxation time brain scores, respectively. B. Regions with stable brain salience of the developmental PLS. Bootstrap ratio is thresholded at ±2.5 as indicated by gradient scale on the right. Anatomical left is left. (TIF) [file pone.0300139.s004.tif]

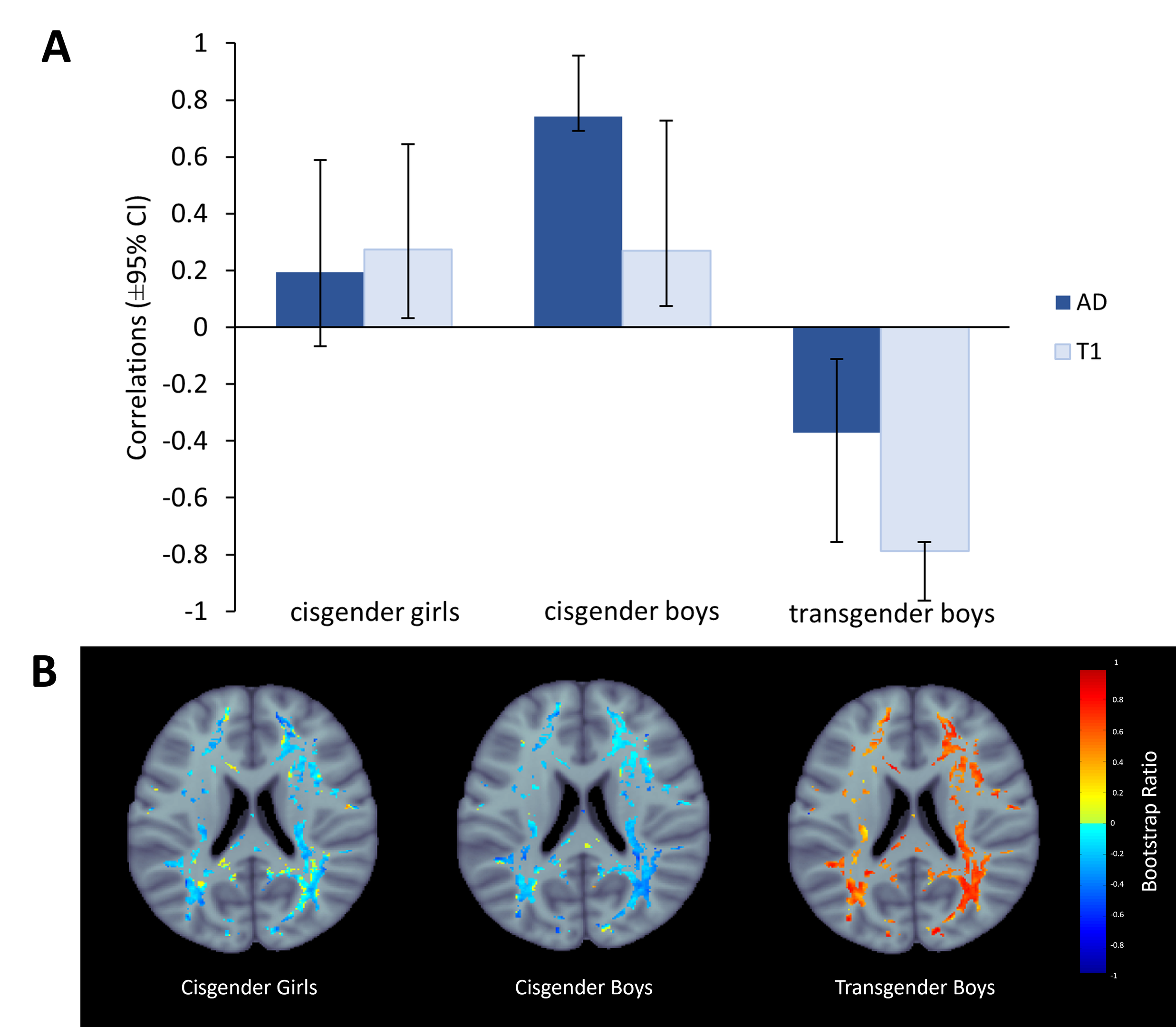

Supplement: S5 Fig — A. LV1 (p = 0.033) accounted for 36.46% of the model covariance. B. Correlation maps of each group depicting differences in strength and direction of T1-estradiol correlation without thresholding the bootstrap ratio. Anatomical left is left. (TIF) [file pone.0300139.s005.tif]
