# Supplementary material for: White matter microstructure in transmasculine and cisgender adolescents: A multiparametric and multivariate study
Source: PLoS One. 2024 Mar 12;19(3):e0300139. doi: 10.1371/journal.pone.0300139 (PMC10931471; doi:10.1371/journal.pone.0300139)
Supplement: S1 Table — (PDF) [file pone.0300139.s006.pdf]

Table S.1. Frequency of self-reported ethnicities of the participants

Ethnicity	Frequency (%)
European/White	69.5
Asian	15.2
East Indian	8.7
Latin American	6.5
Aboriginal	6.5
Other[a]	13

[a]Other inputs included “Canadian,” “Caribbean,” “West Indian,” and “Middle Eastern.”
