# Supplementary material for: White matter microstructure in transmasculine and cisgender adolescents: A multiparametric and multivariate study
Source: PLoS One. 2024 Mar 12;19(3):e0300139. doi: 10.1371/journal.pone.0300139 (PMC10931471; doi:10.1371/journal.pone.0300139)
Supplement: S2 Table — (PDF) [file pone.0300139.s007.pdf]

Correlations	All participants Pearson's *r*	Cis girls Pearson's *r*	Cis boys Pearson's *r*	Trans boys Pearson's *r*
Age				
PDS	0.62**	0.611*	0.833**	0.41
Estradiol	0.14	-0.001	0.63*	0.08
Direction SA	0.01	-0.01	0.39	0.26
Strength SA	0.53**	0.58*	0.87**	0.2
GIDYQ-AA	-0.12	-0.26	0.15	-0.20
Estradiol				
PDS	0.27	0.21	0.51*	0.07
Direction SA	-0.47*	-0.29	0.58*	-0.25
Strength SA	-0.10	0.01	0.57*	-0.37
GIDYQ-AA	-0.12	0.42	0.57*	-0.19

PDS = pubertal development score; SA = sexual attraction; GIDYQ-AA = gender identity/gender dysphoria questionnaire for adolescents and adults

* indicates a significant correlation at $p < 0.05$

** indicates a significant correlation at $p < 0.001$
