# Supplementary material for: White matter microstructure in transmasculine and cisgender adolescents: A multiparametric and multivariate study
Source: PLoS One. 2024 Mar 12;19(3):e0300139. doi: 10.1371/journal.pone.0300139 (PMC10931471; doi:10.1371/journal.pone.0300139)
Supplement: S3 Table — (PDF) [file pone.0300139.s008.pdf]

Cluster	*k* (voxel number)	*p*-value	MNI coordinates (x,y,z)
AD			
1	624	0.02	116, 154, 88
2	115	0.039	71, 162, 91
RD			
1	69597	<0.001	59, 102, 76
MD			
1	69044	<0.01	71, 109, 64
