# Supplementary material for: White matter microstructure in transmasculine and cisgender adolescents: A multiparametric and multivariate study
Source: PLoS One. 2024 Mar 12;19(3):e0300139. doi: 10.1371/journal.pone.0300139 (PMC10931471; doi:10.1371/journal.pone.0300139)
Supplement: S4 Table — (PDF) [file pone.0300139.s009.pdf]

Group	Estradiol	Age (months)	Strength SA	Direction SA	PDS
AD					
Cisgender girls	0.15	**0.54**	0.18	**-0.54**	-0.01
Cisgender boys	**0.7**	**0.73**	**0.69**	**0.53**	**0.60**
Transgender boys	-0.23	0.29	-0.22	**0.77**	-0.46
T1					
Cisgender girls	0.22	-0.08	0.11	-0.28	**-0.42**
Cisgender boys	**0.28**	**0.40**	**0.45**	**0.34**	**0.21**
Transgender boys	**-0.59**	-0.27	-0.27	0.37	-0.45

Significant and stable correlations are in bolded font.
